# ROK study-C (Rainbow of KIBOU study-colorectum): a colorectal cancer survivor cohort study on food, nutrition, physical activity, psychosocial factors and its influences on colorectal cancer recurrence, survival and quality of life in Japan

**DOI:** 10.1186/s12885-018-4830-7

**Published:** 2018-10-04

**Authors:** Yuri Mizota, Yukihide Kanemitsu, Shunsuke Tsukamoto, Dai Shida, Hiroki Ochiai, Seiichiro Yamamoto

**Affiliations:** 1 Division of Health Sociology, Center for Public Health Sciences, National Cancer Center, 5-1-1 Tsukiji, Chuo, Tokyo, 104-0045 Japan; 2 Department of Colorectal Surgery, National Cancer Center Hospital, 5-1-1 Tsukiji, Chuo, Tokyo, 104-0045 Japan

**Keywords:** Colorectal cancer, Survivorship cohort, Diet, Physical activity, Psychosocial factor

## Abstract

**Background:**

Many studies have shown that lifestyle factors such as diet, physical activity are related to the incidence of cancer. However, there are few studies on the association between lifestyle factors and cancer prognosis. To investigate the influence of lifestyle factors and psychosocial factors on prognosis, we started a prospective study of women with breast cancer, the Rainbow of KIBOU study-Breast (ROK Study-B) in 2007. As of February 2018, more than 6300 women have been enrolled, thus making this one of the world’s largest cancer patient cohort studies. Based on the know-how obtained from this study, we started another new cohort study for colorectal cancer patient (ROK Study-C).

**Methods:**

The ROK Study-C is a prospective observational study for colorectal cancer survivors at the National Cancer Center Hospital. Participants fill in several self-administrated questionnaires about lifestyle, psychosocial factors (including posttraumatic growth and benefit finding, support), and quality of life (QOL) 5 times in total: at diagnosis, 3 and 6 months, 1 and 5 years after surgery. CT-scans will be collected to assess body composition and obesity. We also use blood and cancer tissue from the Biobank. The primary endpoint is disease-free survival. The secondary endpoints are overall survival and health-related QOL. The planned sample size is 2000 and the follow-up period is 5 years after the last enrollment.

**Discussion:**

Recruitment began in December 2015 and the study is still ongoing. The ROK Study-C will contribute to improvements in patient prognosis and yield important evidence for colorectal cancer survivorship.

## Background

Colorectal cancer (CRC) is one of the most common cancers in the world. In Japan, CRC is the second most common cancer in both males and females, and the probability of having CRC during one’s lifetime is 10% for men (1 in 10) and 8% in women (1 in 13) [1]. However, the mortality rate tends to decrease as treatments and cancer screening rates improve, and the 5-year relative survival rate is approximately 70% for both men and women [2]. Thus, the number of CRC survivors is steadily increasing.

However, the psychosocial concerns such as anxiety of recurrence remains in survivors, and therefore, in addition to medical treatments, they are also willing to make their own efforts to prevent recurrence, such as changing their diet and levels of physical activity and incorporating complementary and alternative medicine (CAM) into their daily lives [3–7]. For this circumstance, evidence-based lifestyle guidelines are needed.

Many studies have shown that lifestyle factors such as diet, physical activity, and obesity are related to the incidence of cancer, and that it is possible to lower the risk of cancer morbidity by improving lifestyle habits [8, 9]. Since various lifestyle factors are related to cancer morbidity, the possibility that these factors may also affect cancer prognosis has also been considered, and physical activity and obesity, in particular, are expected to be factors related to improvement of prognosis. However, such research has just begun, and sufficient evidence has not yet been obtained [10–12].

Against this backdrop, cohort studies investigating cancer patients are being started. We also started a breast cancer survivorship cohort study titled the Rainbow of KIBOU (ROK) Study-B in November 2007 [13]. In addition to diet, physical activity, CAM use, we focused on psychosocial factors such as support, hope, posttraumatic growth, benefit finding, and social participation. A self-administered questionnaire is delivered to patients enrolled and their responses are regarded as baseline data. Blood samples are collected at each year for 5 years and tissue samples are collected at surgery. As of August 2017, baseline data has been obtained from over 6000 breast cancer patients, thus making this the world’s largest cancer patient cohort. Participants are still being enrolled.

Based on the experience and know-how obtained from this study, we started another new cohort study for CRC patient (ROK Study-C).

The ROK Study-C will reveal the relationship between diet, physical activity, obesity, CAM use, psychosocial factors, blood biomarkers, and genetic polymorphisms, and CRC prognosis such as recurrence, mortality, secondary cancers, and QOL. By doing so, this study will contribute to improvements in patient prognosis. In addition, this study will make it possible to provide important evidence to patients, their families, and their health care providers etc., and lead to the development of lifestyle guidelines for cancer survivorship.

In this article, we describe the formation of the ROK Study-C.

## Methods/design

The ROK Study-C is a prospective observational cohort study aiming to examine the influence of lifestyle such as diet, physical activity, obesity, CAM use, psychosocial factors, etc., on the CRC prognosis such as recurrence, survival, and QOL. In addition to several self-administrated questionnaires for data collection, we use blood samples and cancer tissues provided by the Biobank at the National Cancer Center (NCC) Hospital with their consent.

The planned number of participants is 2000. The start of the study period is the date of acquisition of Institutional Review Board (IRB) approval, the enrollment period is 7 years from the enrollment of the first participant, and the follow-up period is 5 years from the enrollment of the last participant.

### Recruitment

The eligibility criteria include men and women with primary CRC, in stage I-III, between 20 and 80 years, anticipating surgery at the NCC hospital. People who appeared not to be ethnically Japanese will be excluded from the study.

Eligible patients receive an informed consent form about this study from their treating physician shortly after diagnosis during a clinical visit before surgery. Among patients meeting the eligibility criteria, those who provide informed consent for participation in this study are considered eligible.

### Data collection

**Fig. 1:**
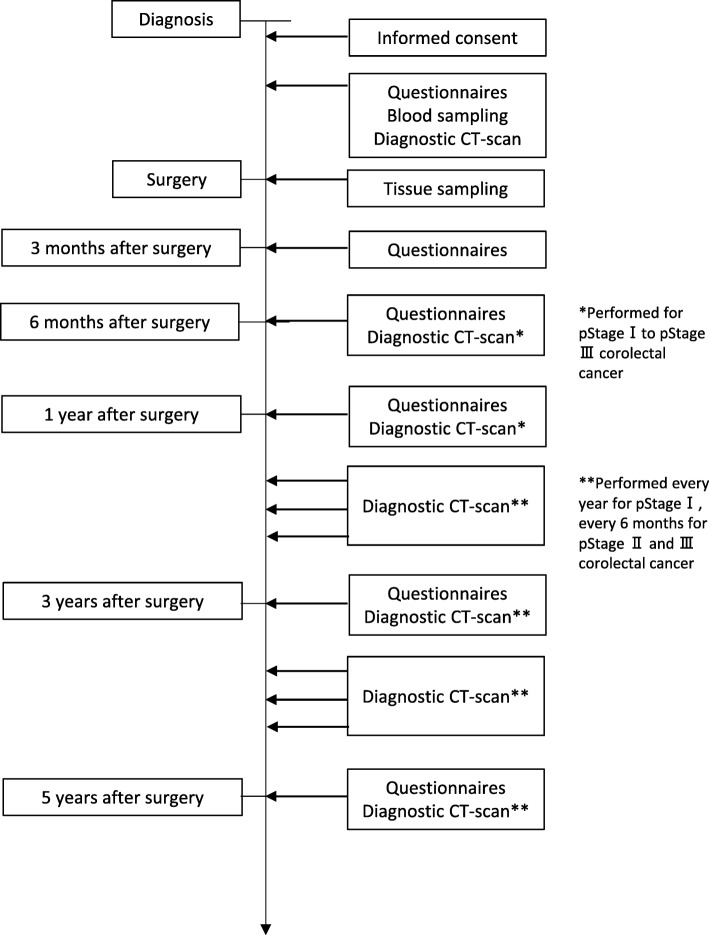
Overview and design of the ROK Study-C

An overview of this study and the data collection schedule are described in Fig. 1.

Participants are asked to fill in several self-administrated questionnaires about lifestyle, psychosocial factors, and quality of life (QOL) 5 times in total: at diagnosis, 3 months, 6 months, 1 year and 5 years after surgery. At the first survey, participants are asked about their typical lifestyle habits before diagnosis, and after surgery, they are asked about their typical lifestyle habits at each time point. Questionnaires are based on the same questionnaire used for ROK Study-B. Additionally, we use blood samples collected once before surgery and cancer tissues from the NCC Biobank.

#### Demographic and health characteristics

Self-administrated questionnaires collect information on demographics (education, marital status, number of children, living situation, income, occupation and social activities), height, body weight, history of body weight, smoking history, history of medication including Non-Steroidal Anti-Inflammatory Drugs (NSAIDs), developmental history, family health history, and for women, menopausal status, menstrual and reproductive history and hormone use.

#### Diet

Although the etiological role of diet such as alcoholic beverages, red meat processed meat in CRC has been recognized [9], much less is known the association between diet and CRC prognosis. In a few study accessed the relationship between diet or nutrition and CRC prognosis, it was shown that the risk of recurrence or death increases with ‘Western diet’ (meals high in meats, fats, refinded grains, and desserts) [14], and the risk is lowered in people with high blood vitamin D concentrations [15]. However there is insufficient evidence to confirm these findings [11]. Therefore, in ROK Study-C, we measured participants’ food intake including meat and vegetables, nutrients such as vitamins, and dietary patterns, and investigated the relationship between these factors and prognosis. In this study, a food frequency questionnaire (FFQ) is used to assess the average intake of 147 food and beverage items over the previous year [16]. For most food items, participants were asked about consumption frequency and their usual portion size. The validity and reproducibility of the FFQ had already been established as reasonable [17–19].

#### CAM use

In addition to diet and exercise, many patients are interested in and actively practice CAM. This includes the use of oral and topical agents and health-promoting methods such as acupuncture, moxibustion, and yoga, which differ from health insurance-supported practice. Although approximately 50% of cancer patients utilize CAM in Japan [20], there is insufficient evidence regarding its survival efficacy [7, 21]. The use of CAM is assessed with a self-administrated questionnaire based on previous studies. Participants are asked at each survey point about the use (including before diagnosis), total length of use, frequency, cost, and duration, reasons for starting and withdrawing from each CAM [13].

#### Physical activity

Low levels of physical activity is a convincing risk factor for CRC [9]. Further in relation to the prognosis of CRC, the level of physical activity is one of the most promising factors for improving prognosis [11, 12, 22]. In addition, the increase in the amount of physical activity is also important in terms of improving the QOL of cancer patients and preventing other cancers and cardiovascular diseases [12], so it may lead to reduce all-cause mortality of CRC survivors.

In ROK Study-C, a self-administrated questionnaire previously validated during the JPHC Study is used to assess physical activity [23]. Prior to surgery, participants are asked about their typical level of physical activity during the year prior to diagnosis and again 1 year and 5 years after surgery.

#### Obesity

Since obesity is also a convincing risk factor for CRC [9], to prevent overweight may improve the prognosis of CRC survivors. Previous studies have shown a relationship between higher body mass index (BMI) and body fatness before or at the time of diagnosis and a higher all-cause mortality or CRC recurrence or CRC-specific mortality, however, results were not consistent [11].

While BMI is not a valid measure for fat distribution or body composition, research using other markers such as waist and hip circumference and computed tomography (CT) scan [24, 25].

**Fig. 2:**
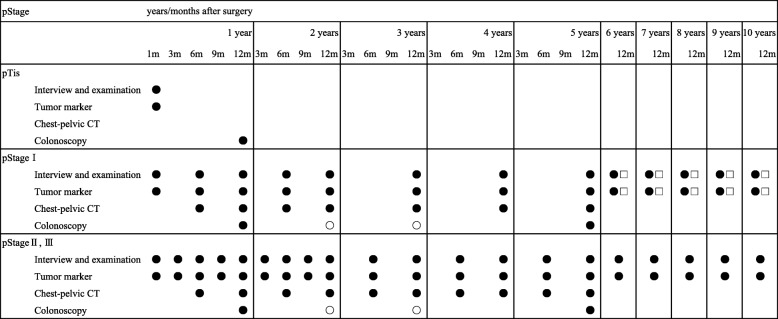
A surveilance schedule after curative resection accordning to pathological cracifications. Legend: ●: Performed for pStage I to pStage III corolectal cancer, ○: Perfomed for rectral cancer, □: Perfomed for pMP

In ROK Study-C, in addition to BMI, data are collected via CT-images for the assessment of body composition. CT-images are retrieved from medical records of all participants as shown in Fig. 2.

#### Other lifestyle factors

Participants were also asked about meals and taste preferences, bathing, sleeping, dental health, and use of the Internet.

#### Psychosocial problems

CRC survivors experience many psychosocial challenges and concerns during not only pre-operative but also post-operative period. Psychosocial issues reported in CRC survivors include depression, anxiety, disturbance in body image, avoiding coping, hopelessness and stress related to physical and psychosocial changes [26–33]. There is a lack of research exploring the relationship between these psychosocial problems and CRC prognosis. Some previous studies have reported an association between hopelessness, coping style, and recurrence, whereas others have found no association, and consistent and reliable results have not been obtained [33].

In ROK Study-C, psychosocial problems are assessed at each survey point. The Japanese version of Hospital Anxiety and Depression Scale (HADS) is used to measure the level of depression and anxiety [34]. To assess the stress, a self-administrated questionnaire based on previous studies and interviews of cancer survivors is used. Participants are asked the existence and degree of 14 stressful conditions from cancer [13].

#### Psychosocial well-beings

Our goal of supporting cancer survivors is not absence of psychosocially illness but presence of psychosocially wellness. So ROK Study-C focuses on well-beings of CRC survivors. In recent years, conceptions of psychosocial well-being such as ‘*ikigai’* (‘something that makes one’s life seem worth living’, described in detail below), ‘hope’ (not ‘hopeless’ but ‘hope’) [35, 36], ‘posttraumatic growth (PTG) and benefit finding (BF),’ and social support are beginning to be noticed in relation to adaptation to adverse circumstances such as illness and improvement of QOL.

*Ikigai*, in Japanese, is used to describe a concept closer to ‘a purpose in life’, ‘a reason to live’ or ‘something that makes one’ life seem worth living’, and includes various factors such as family, hobbies/enjoyment, connection with friends/community, social participation, etc. [37]. Having *ikigai* only enriches life, but also draws attention to health outcomes. In Japanese studies, having *ikigai* demonstrated lower risk of psychosocial problems such as depression and lower mortality from all cause, cerebrovascular diseases and ischemic heart disease [38–42]. However, the associations between cancer mortality or incidence and *ikigai* were inconsistent [41–44]. Though there is no term fully comparable in English, the concept of *ikigai* is becoming understood around the world. In ROK Study-C, a self-administrated questionnaire based on previous studies and interviews of cancer survivors is used to assess *ikigai* [13, 38]. Participants are asked the existence of 7 items of *ikigai* and space is available to report the *ikigai* that are not specified on the questionnaire.

Hope is one of the most commonly used indicators of subjective well-being. Hope is one of the essential elements of life that forms the basis of life in every stage of peoples’ lives, including cancer survivors [35, 36]. Hope helps people find meaning in struggle and maintain a will to endure even in adversity, and is thought to be related to adaptability and coping strategies to adapt to difficulty [35, 36, 45, 46]. A high level of hope plays an important role in maintaining one’s physical condition in cancer patients as well, and has been shown to be strongly associated with adaptation to cancer [47, 48]. However, few longitudinal studies have examined the association between hope and prognosis in CRC survivors. To evaluate the level of hope, we employ the Japanese version of Herth Hope Index (HHI) [49].

Exposure to stressful and traumatic events can have severe and chronic psychological consequences. Researches on adversities have long been focused on the negative influence of these events on changes in peoples’ lives [50–52]. These theories have made a great contribution to understanding the difficulties of people in times of adverse life events. However, in the past 20 years, from the criticism of emphasizing only negative influences, researches have also aimed to consider the positive impact of adversities. Many studies focused on positive psychology have reported positive changes such as posttraumatic growth (PTG) and benefit finding (BF) in people who have experienced various traumatic events such as disease, disaster, bereavement, crime, etc. [53–56]. Such positive changes are products brought about in the process of adaptation to an adversity and can also be a coping strategy for adaptation. Thus, they play important role in the cognitive adaptation theory to adversity, and expect to reconstruct lives of people in adversities. A few studies have found that PTG and BF were experienced relatively large proportion of CRC survivors [57–59], however, prevalence of PTG and BF in long-term CRC survivor, factors that develop positive changes, and the association between positive changes and CRC prognosis have not been sufficiently examined. In this study, a self-administrated questionnaire based on previous studies and interviews of cancer survivors is used to assess PTG and BF [13, 38].

In ROK Study-C, psychosocial well-beings are assessed at each survey point.

#### Blood and cancer tissue

Biomarkers from blood samples will be investigated for the association with prognosis and treatment effect. The following is the list of candidate substances to be measured: endogenous hormones (estrone, estradiol, sex-hormone binding globulin, androgen, etc.), insulin and insulin-like growth factor (C-peptide, IGF-1, IGFBP-3, IGFBP-1, etc.), adipocytokines (adiponectin, leptin, etc.), markers of inflammation (C-reactive protein, etc.), nutrients (isoflavones, folate, 25-hydroxyvitamin D, etc.), and other substances (markers of oxidative stress, etc.). Germline mutations will be used for the association with prognosis and toxicity of drugs. The following is the list of candidates genes to be assessed: genetic polymorphisms of hormone synthesized and metabolized genes, hormone receptor genes, genes related to insulin-like growth factor, genes related to one-carbon metabolism, vitamin D receptor genes, genes related to oxidative stress, and genes related to the metabolism of therapeutic agents. Tissue biomarkers will be used for sub-classification by molecular subtype to investigate the exposure-tumor subtype interaction.

### Endpoints

Primary endpoint is disease-free survival (DFS) and secondary endpoints are overall survival, health-related QOL (HRQOL), toxicity, surgical complications (sexual dysfunction, urinary tract problems, anal dysfunction), tumor response to drug, and secondary cancer.

### Outcome ascertainment

#### Recurrence and survival

Follow-up in the daily practice of NCC Hospital is based on the Japanese guidelines [60], as shown in Fig. 2. In cases involving hospital transfers or deaths, inquiries will make with the receiving hospital or be linked with data contained in the cancer registry.

#### HRQOL

A number of instruments are used in ROK Study-C to measure various dimension of QOL at each survey points. The MOS 36-item Short Form Survey (SF-36) [61] is used as a comprehensive QOL. Cancer-related QOL is assessed by the European Organization for Research and Treatment of Cancer QLQ-C30 (EORTC QLQ-C30) [62] and Colorectal cancer 29 (CR-29) [63]. Wexner’s Scale [64], an index of postoperative anal function is used to evaluate bowel movement function. International prostate score (I-PSS) [65] and overactive bladder symptom score (OABSS) [66, 67] are used to evaluate urinary function.

For sexual function in men, a 15-item version of the International Index Erectile Function (IIEF) [68] score is used. For women, the Female Sexual Function Index (FSFI) [69] is used. The FSFI was developed as an evaluation scale for multidimensional and objective measurement of female sexual function, and it has been translated into approximately 30 languages and is widely used. Although the original version of the FSFI contains questions regarding participants’ condition over the past month, the Japanese version considered the frequency of sexual intercourse in Japan and this period was changed to the past 3 months, and the validity of this change was validated [70].

#### Adverse events

The Common Terminology Criteria for Adverse Events (CTCAE) ver. 4.0, Japan Clinical Oncology Group/Japanese Society of Clinical Oncology (JCOG/JSCO) version is used to evaluate adverse events, and the evaluation will be carried out in accordance with NCC Hospital postoperative treatment guidelines.

### Sample size

Sample size was decided to investigate the exposure effects on the disease-free survival (primary endpoint). Based on the historical data of NCC Hospital, we estimated 5 year-DFS of unexposed group as 60–90%. Number of needed events and sample size are shown in Table 1 for 7-year accrual and additional 5-year follow-up. We decided sample size as 2000 in order to detect 5% increase in exposure group with two-sided alpha level of 0.05 and a power of 80%.

**Table 1:** Number of necessary events and sample size to obtain 80% statistical power

Scenario				Necessary number in two group	
5-year Disease Free Survival					
Unexposed group	Exposed group	Difference between exposed to unexposed	Hazard Ratio of exposed to unexposed	events	sample size
90%	95%	5%	0.49	67	550
85%	80%	5%	0.65	173	862
80%	85%	5%	0.73	318	790
75%	80%	5%	0.78	491	1152
70%	75%	5%	0.81	684	1646
65%	70%	5%	0.83	884	1842
60%	65%	5%	0.84	1084	2002

Necessary numbers are calculated with scenario where 7-year accrual and 5-year follow-up

### Data analysis

Following the review of WCRF/AICR Breast Cancer Survivorship [7], we plan to analyze the effect on prognosis of lifestyle before diagnosis and lifestyle after 1 year from the diagnosis, respectively. Exposure effects will be estimated using regression type models controlling confounding factors such as Cox, logistic and linear regression according to the types of the outcome variables.

## Discussion

To investigate the influence of lifestyle factors, psychosocial factors on prognosis, we started a prospective study of women with breast cancer, the Rainbow of KIBOU study-Breast (ROK Study-B) in 2007. As of February 2018, more than 6300 women have been enrolled, thus making this one of the world’s largest cancer patient cohort studies. Based on the experience and know-how obtained from ROK Study-B, we started another new cohort study for colorectal cancer patient (ROK Study-C). Recruitment of ROK Study-C began in December 2015 and there are 575 participants enrolled as of February 23, 2018, and the enrollment is still ongoing.

To support survivorship of CRC patients, the ROK Study-C aims to assess the influence of various factors (diet, physical activity, CAM use, smoking, drinking, psychosocial factors, etc.) on prognosis (recurrence, mortality, etc.) and long term HRQOL. In addition to the lifestyle, the ROK Study-C also focuses on psychosocial factors. One of the main objectives of the ROK Study-C is to investigate the psychosocial factors influencing survivorship, including the *ikigai* (a concept closer to ‘a purpose in life’, ‘a reason to live’ or ‘something that makes one’ life seem worth living’), hope, positive change such as PTG and BF, job, social activities, and support that are important to living with cancer. The ROK Study-C will clarify the importance of these factors in terms of their association with prognosis and long term QOL. It will provide evidence-based guidance about how to improve prognosis and QOL. There are almost no cohort studies that collect data on these factors.

So far, although the effect of lifestyle and psychosocial factors on CRC prognosis is not known, survivors make their own efforts to prevent recurrence, such as changing their diet and levels of physical activity and CAM use etc. Because evidence-based guidance is eagerly awaited by patients, clarifying the factors that are truly effective in improving the prognosis will lead to prompt and widespread adoption and impact. The name of this study, “ROK” was decided together with cancer survivors. “ROK” is an acronym standing for “Rainbow of Kibou.” Kibou is the Japanese word for “hope.” We are conducting this study in the hope that the results will help fulfill survivors’ wishes. The ROK Study will contribute to improvements in patient prognosis and yield important evidence for cancer survivorship.

## Abbreviations

BF: Benefit finding
BMI: Body mass index
CAM: Complementary and alternative medicine
CR-29: Colorectal cancer 29
CRC: Colorectal cancer
CT: Computed tomography
CTCAE: Common Terminology Criteria for Adverse Events
DFS: Disease-free survival
EORTC QLQ-C30: European Organization for Research and Treatment of Cancer QLQ-C30
FFQ: Food frequency questionnaire
FSFI: Female sexual function index
HADS: Hospital anxiety and depression scale
HRQOL: Health-related quality of life
IGF: Insulin-like growth factor
IGFBP: Insulin-like growth factor binding protein
IIEF score: International Index Erectile Function score
I-PSS: International prostate score
IRB: Institutional Review Board
JCOG/JSCO: Japan Clinical Oncology Group/Japanese Society of Clinical Oncology
NCC: National Cancer Center
NSAIDs: Non-Steroidal Anti-Inflammatory Drugs
OABSS: Overactive bladder symptom score
PTG: Posttraumatic growth
QOL: Quality of life
ROK Study-B: Rainbow of KIBOU Study-Breast
ROK Study-C: Rainbow of KIBOU Study-colorectum
SF-36: The MOS 36-item Short Form Survey
WCRF/AICR: World Cancer Research Fund/American Institute for Cancer Research
